# Isolated Splenic Cold Abscesses with Perisplenic Extension: Treated Successfully without Splenectomy

**DOI:** 10.1155/2017/9864543

**Published:** 2017-08-20

**Authors:** Mohan Khadka, Ravi Pradhan

**Affiliations:** ^1^ADK Hospital, Malé, Maldives; ^2^Institute of Medicine, Tribhuvan University Teaching Hospital, Kathmandu, Nepal

## Abstract

Splenic tuberculosis (TB) in the form of multiple splenic cold abscesses with perisplenic extensions is a rare disease, especially in an immunocompetent host. It demonstrates diagnostic complexity, which makes identification of the disease difficult. We report a case of an immunocompetent adult male who presented with fever, pain in the left lower chest, decreased appetite, and significant weight loss. On physical examination, he had tenderness in the left lower infra-axillary region and Traube's space dullness without palpable spleen. Ultrasound-guided aspiration of the abscess fluid revealed* Mycobacterium tuberculosis* (MTB) by polymerase chain reaction (PCR). No primary focus of the infection was detected in the lungs or any other organs. The patient was successfully treated with antitubercular therapy (ATT).

## 1. Introduction

Tuberculosis (TB) is more common in a developing country like Nepal than in the developed world. It continues to be a major health hazard, in spite of notable advances in diagnosis and treatment [1]. TB may present as a pulmonary or extrapulmonary disease. Of all organs, lungs are the predominantly affected organ. Tuberculosis of the spleen is rare, except as a part of miliary tuberculosis [2]. TB of the spleen without any other detectable primary site, as in our report, is considered primary tuberculosis of the spleen. Isolated splenic tuberculosis may not have the characteristic symptoms of tuberculosis; therefore, it is likely to be misdiagnosed as splenic malignancy, abscess, lymphoma, rheumatic fever, or other conditions [3].

## 2. Case Report

A 22-year-old nonsmoker, nonalcoholic male from a middle-class family in the midwestern part of Nepal was referred to our tertiary center with intermittent fever, pain in the left lower chest, occasional nausea and vomiting, anorexia, and weight loss of three-week duration. Symptoms did not improve with two weeks of broad-spectrum oral and then parenteral antibiotics at a local hospital. Fever was associated with chills and rigors. Left lower chest pain was continuous with radiation to the left shoulder. There was no associated cough, shortness of breath, altered bowel or bladder habits, jaundice, headache, vomiting, joint pain, or any skin nodules. There was no history of drug abuse or sexual promiscuity. There was no history of diabetes mellitus and no past or family history of TB.

**Figure 1:**
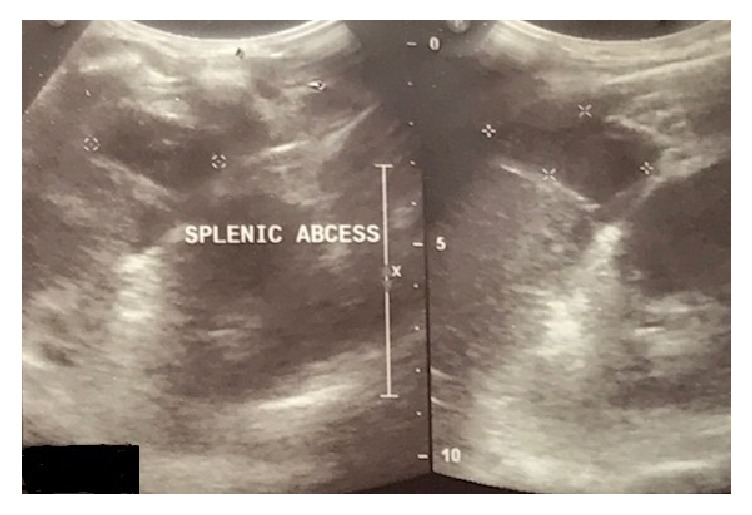
Ultrasound of the abdomen showing multiple hypoechoic splenic and perisplenic collections.

**Figure 2:**
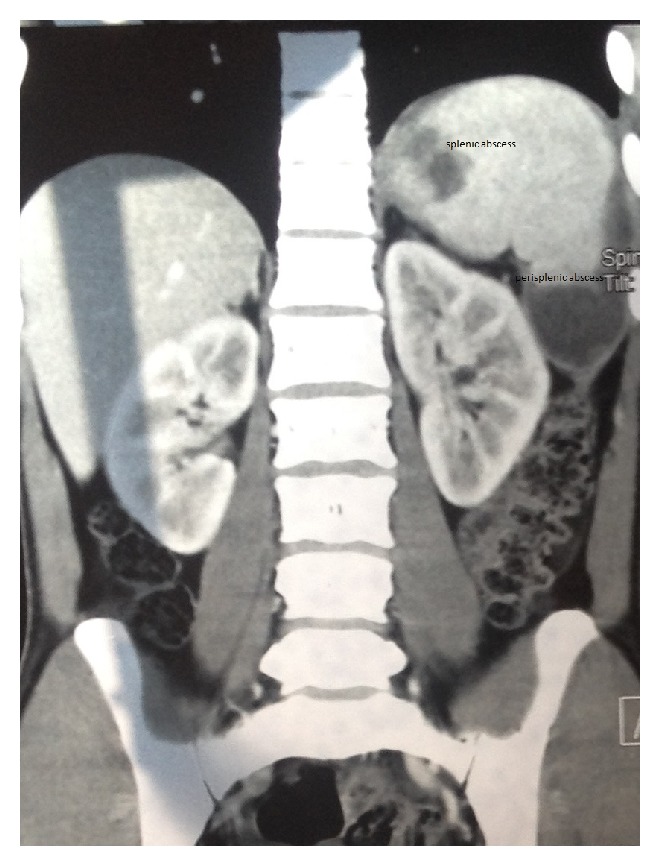
Contrast-enhanced computed tomography of the abdomen showing multiple hypodense lesions in and around the spleen.

On examination, the patient's body temperature was 102°F, pulse was 100 beats per minute, and respiratory rate was 16 breaths per minute and regular. Chest examination showed tenderness in the left infra-axillary region and normal bilateral vesicular breath sounds without any additional sounds. Cardiovascular examination revealed normal heart sounds without murmur. Abdominal examination showed a soft, tender left upper quadrant with Traube's space dullness; however, the spleen and liver were not palpable. Complete blood count, liver and renal function tests, posteroanterior chest X-ray, and echocardiography were normal. Erythrocyte sedimentation rate (ESR) using Wintrobe's method was elevated at 58 mm per hour. Purified protein derivative (PPD) was 12 mm. Sputum samples on three consecutive days were negative for acid-fast bacillus (AFB). Spot serological tests for human immunodeficiency virus, hepatitis B, and hepatitis C were nonreactive. Three consecutive bacterial blood cultures and a urine culture showed no growth. Serologic tests for malaria, leptospirosis, brucellosis, and leishmaniasis were negative. Ultrasound of the abdomen (Figure 1) revealed a 5.3 × 4.0 × 3.5 cm hypoechoic lesion at the lower pole of the spleen that was in communication with a similar lesion of an approximately 40 ml collection at the perisplenic area. Likewise, a 3.9 × 3.0 × 2.8 cm collection at the superior pole of the spleen was in communication with a similar collection at the perisplenic area. Contrast-enhanced computed tomography of the abdomen (Figure 2) showed two perisplenic hypodense collections of 6.9 × 5.1 × 3.2 cm at the inferior pole and 5.2 × 4.0 × 3.0 cm at the superior pole of the spleen having connections with similar splenic parenchymal collections, suggestive of multiple splenic abscesses with perisplenic extensions. The patient did not improve with three weeks of empiric parenteral broad-spectrum antibiotics.

Diagnostic and therapeutic ultrasound-guided aspiration removed approximately 30 ml of pus which was sent for microbiological and molecular examination for MTB. The pus was negative for AFB staining; however, PCR for MTB was positive. Gram's stain revealed no bacteria, and routine 72-hour bacterial culture showed no microorganism growth.

**Figure 3:**
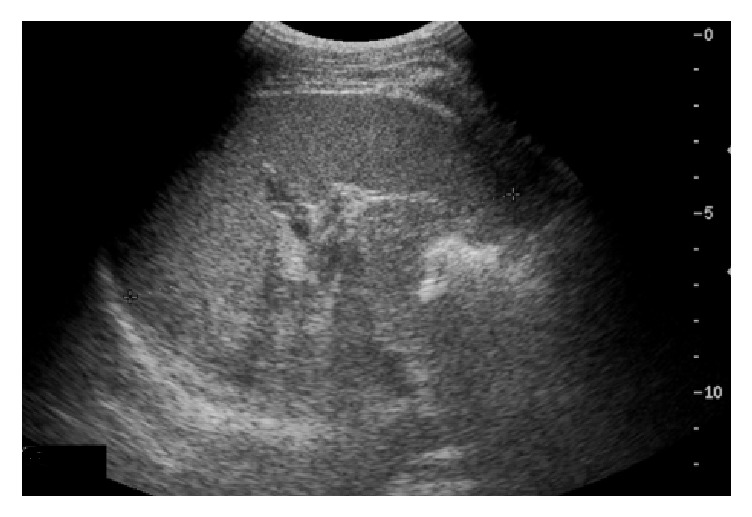
After six months of ATT, ultrasound showed a normal spleen.

Diagnosis of primary splenic tuberculosis was made, and as per directly observed treatment, short course (DOTS), ATT category I was initiated for 6 months. One week after starting ATT, the patient improved clinically with fever resolution and an increase in appetite. The patient was then discharged on ATT. On follow-up, the patient had clinically improved and gained weight. Serial ultrasounds of the abdomen showed a gradual decline in the abscess sizes. Upon completion of six months of ATT, the patient was clinically asymptomatic and had normal ESR, and ultrasound of the abdomen (Figure 3) showed normal spleen.

## 3. Discussion 

Clinically, TB might present as a pulmonary or extrapulmonary disease. Splenic TB occurs in two forms. The first form presents itself as a part of miliary TB, especially in immunocompromised patients, which is common. Its treatment includes classic ATT, which might improve the patient's overall immunity. This form rarely requires surgical intervention [4]. The second and less common form of splenic TB is primary involvement of the spleen, as in our patient. Many reported cases of splenic tubercular abscess are found to have underlying HIV infections [1]. However, there are only sporadic case reports of splenic TB in immunocompetent patients [5]. Although Winternitz in 1912 categorized splenic TB as either a primary or a secondary form, some scholars insist that all patients with splenic TB are secondary to previous infection of tubercle bacilli in other organs [6]. Our patient was apparently immunocompetent and had no past or family history of TB. A case of splenic TB, reported by Ho and colleagues, presented with fever and weight loss but without pain in the left lower chest and hypochondrium region [7]. However, in our case, the patient presented with fever, weight loss, and pain in the left lower chest.

Diagnosis of isolated splenic TB is difficult and often delayed because of imprecise clinical manifestations. In almost all of the reported cases, the definitive diagnosis is generally made with splenic biopsy or postsplenectomy specimens and is rarely made on the basis of only imaging with or without fine needle aspiration [8]. In our case, diagnosis was made by radiological findings, both abdominal ultrasound and CT, followed by aspiration of the abscess. AFB smear of the pus was negative; however, PCR confirmed the presence of* Mycobacterium tuberculosis*. Tubercular infection can be histopathologically identified by typical caseating granuloma composed of epitheloid cells and Langhans giant cells, but these findings cannot differentiate between an infection due to* Mycobacterium tuberculosis* and one caused by nontubercular mycobacteria. Diagnosis often requires a specimen; however, splenic biopsy has a high risk of complication, and splenectomy poses a high risk and is unnecessary.

A case report by Zhan and colleagues described the histopathological diagnosis of* Mycobacterium tuberculosis* of the spleen using CT-guided fine-needle aspiration, precluding the need for a splenectomy [3]. In our study, we were able to make a diagnosis using PCR of a specimen obtained by ultrasound-guided aspiration. The advantages of PCR for diagnosis of MTB are its rapidity, sensitivity, and high specificity [9].

As with treatment of pulmonary tuberculosis, treatment of splenic TB must be carried out in accordance with the following principles: timely treatment in combination with regular and proper monitoring through the whole course whether or not splenectomy is performed. Splenic tuberculosis requires six months of treatment. Standard ATT should be taken preoperatively and postoperatively if splenectomy is carried out [3]. There is no case report where primary multiple splenic tubercular abscesses with perisplenic extensions were treated successfully with therapeutic ultrasound-guided aspiration of perisplenic abscess followed by ATT, which precluded the need for splenectomy as in our case.
